# Vertical bone regeneration using rhBMP-2 and VEGF

**DOI:** 10.1186/s13005-017-0146-0

**Published:** 2017-06-07

**Authors:** Lara Schorn, Christoph Sproll, Michelle Ommerborn, Christian Naujoks, Norbert R. Kübler, Rita Depprich

**Affiliations:** 1 Department of Oral-, Maxillo- and Plastic Facial Surgery, Heinrich-Heine-University Duesseldorf, Moorenstr. 5, 40225 Duesseldorf, Germany; 2 Department of Operative and Preventive Dentistry and Endodontics, Heinrich-Heine-University Duesseldorf, Moorenstr. 5, Duesseldorf, 40225 Germany

**Keywords:** Vertical bone regeneration, Cytokines, rhBMP-2, VEGF, Tissue engineering

## Abstract

**Background:**

Sufficient vertical and lateral bone supply and a competent osteogenic healing process are prerequisities for the successful osseointegration of dental implants in the alveolar bone. Several techniques including autologous bone grafts and guided bone regeneration are applied to improve quality and quantity of bone at the implantation site. Depending on the amount of lacking bone one- or two-stage procedures are required. Vertical bone augmentation has proven to be a challenge particularly in terms of bone volume stability. This study focuses on the three dimensional vertical bone generation in a one stage procedure in vivo. Therefore, a collagenous disc-shaped scaffold (ICBM = Insoluble Collagenous Bone Matrix) containing rhBMP-2 (Bone Morphogenetic Protein-2) and/or VEGF (Vascular Endothelial Growth Factor) was applied around the coronal part of a dental implant during insertion. RhBMP-2 and VEGF released directly at the implantation site were assumed to induce the generation of new vertical bone around the implant.

**Methods:**

One hundred eight titanium implants were inserted into the mandible and the tibia of 12 mini pigs. Four experimental groups were formed: Control group, ICBM, ICBM + BMP-2, and ICBM + BMP-2 + VEGF.

After 1, 4 and 12 weeks the animals were sacrificed and bone generation was investigated histologically and histomorphometrically.

**Results:**

After 12 weeks the combination of ICBM + rhBMP2 + VEGF showed significantly more bone volume density (BVD%), a higher vertical bone gain (VBG) and more vertical bone gain around the implant (PVBG) in comparison to the control group.

**Conclusion:**

By using collagenous disc-shaped matrices in combination with rhBMP-2 and VEGF vertical bone can be generated in a one stage procedure without donor site morbidity. The results of the presenting study suggest that the combination of rhBMP-2 and VEGF applied locally by using a collagenous carrier improves vertical bone generation in vivo. Further research is needed to establish whether this technique is applicable in clinical routines.

## Background

Successful osseointegration of dental implants depends on implant stability, quality and quantity of alveolar bone [1], and bone-to-implant contact [2]. Lower quality or quantity of residual bone can be caused by trauma, systemic or local illnesses or local atrophic processes [3] eventually resulting in the need for bone augmentation. Various studies showed that vertical bone regeneration in particular remains a challenge [4–7]. At posterior regions of the upper jaw a sinus floor augmentation procedure is suitable to regain bone height whereas in the lower jaw there is no such option [8]. In cases where the remaining bone is still 5–8 mm in height mini implants can be an option nowadays but sometimes there even is not sufficient bone for the use of those [9, 10].

Several augmentative techniques and materials have been described for bone regeneration. Autogenous, allogenic, xenogenic or alloplastic onlay or inlay bone grafts can be used for horizontal or vertical bone regeneration. Autogenous bone is the most commonly used and current gold standard [11–14], especially in combination with the tent pole technique (were the periosteum is tented up for bone to grow underneath) [15, 16]. A recent meta-analysis reviewed the use of grafting materials for alveolar ridge augmentation in combination with implant placement. Troeltzsch et al. showed that in terms of vertical bone augmentation autogenous bone blocks harvested from the iliac crest and the calvarium seem to be the only ones capable of gaining larger bone volumes [17]. The main disadvantages of bone grafts are donor site morbidity, limited supply, and possible postoperative complications [18]. When it comes to quality of life after surgery, patients’ discomfort appears to be significantly higher when autogenous bone grafts (especially iliac crest grafts) are used in comparison to other augmentative techniques or materials [6, 17, 19–23]. Bone grafts often cause insufficient bone consolidation and sometimes are limited in size (e.g. space at implantation site is limited due to obligatory gingival coverage) [8]. Screws or other fixation devices might be necessary to keep the graft in place [7]. Furthermore, when used without a membrane technique, there might be fibrous encapsulation of the graft and as a consequence no sufficient bone-to-implant contact [8, 24].

A different approach is guided bone regeneration (GBR) which makes use of occlusive membranes preventing soft tissue ingrowth into the bone thereby allowing osteogenic cells originating from the adjacent bone to immigrate the restoration site [13, 25]. In addition, there are combinations of bone substitutes and membrane techniques [26]. Small volume vertical defects can be augmented by particulate grafting materials. If no skeletal scaffold is available, the combination with titanium meshes seems to be most beneficial for three-dimensional stability [17]. GBR has led to promising results in the past [8, 27–29]: there is unlimited supply and no need of a donor site. Nevertheless, membranes tend to collapse leaving the problem of bone volume stability. Non resorbable membranes or titanium meshes have to be removed in a second surgery. As in any other surgical procedure, infections, tissue inflammation and wound dehiscence have been reported as complications [8, 27].

Distraction osteogenesis is another option for vertical bone gain [13, 30]. Advantages of distraction osteogenesis are soft tissue expansion simultaneously with bone growth and again no donor site morbidity compared to the harvest of autogenous bone [8, 31]. Disadvantages are the long duration of the treatment, high relapse rates and possible post-operative complications such as early, delayed or completely absent bone consolidation, nerve injury and infection [32]. A minimum of bone height and stability is necessary for device application which usually precludes the use of this technique in the severely atrophic jaw [8]. Furthermore, several surgical procedures are required for installation and removal of the distraction device, and at least implant insertion [8, 33].

All of the above mentioned options for bone augmentation are technically highly demanding and depend on good manual skills of the surgical staff [34]. Depending on the augmentation techniques several months are required for bone consolidation and additional surgery might be necessary to finally insert the implant and/or remove the non resorbable materials/devices. Postoperative complications (e.g. infection, wound dehiscence, nerve injury, bleeding) or insufficient bone formation might occur [9]. GBR, onlay grafts and alveolar ridge distraction seem to deliver stable results of bone regeneration after up to 5 years but overall there is very little reliable data to show whether those approaches are successful over time (>10 years) [9, 35].

Since bone tissue engineering holds the promise to provide an alternative to all these above described techniques, research on this topic has become increasingly popular over the last years. Along with mesenchymal and embryonal stem cells, cytokines and growth factors blaze a trail to bone augmentation [36]. The BMPs constitute a family of proteins with the ability to initiate osteoblastic differentiation [37] and have proven to enhance osteoinductive characteristics of bone graft substitutes (e.g. in combination with autogenous bone blocks or collagenous sponges) [38–43]. rhBMP-2 in particular has shown the highest osteoinductive potential of the BMP family [44]. VEGF–A promotes angiogenesis by proliferation and migration of endothelial cells and regulates vasculogenesis [45, 46]. Furthermore, it influences osteoblastic differentiation and plays an important role in early and late enchondral ossification due to resorption of cartilage and promotion of angiogenesis [47]. It has proven to be important for craniofacial and mandibular ossification in particular [48]. By combining VEGF and rhBMP-2 angiogenesis and blood supply can be increased and formation of new bone can be enhanced [44].

The aim of this study was to generate vertical bone growth with the help of a disc-shaped collagenous scaffold containing rhBMP-2 and VEGF placed around the coronal part of the implant during insertion. The following advantages were postulated:

1. Sufficient volume stability and adequate bone consolidation are ensured by application of rhBMP-2 and VEGF directly on implantation site.
2. No membranes, screws or titan meshes are necessary to hold the augmentative materials in place or prevent soft tissue from ingrowth.
3. In comparison to other studies and techniques no consecutive surgery is necessary in order to remove foreign materials [49, 50].
4. The implant can be set directly at its ideal position. Simultaneously, the implant is able to integrate into the residual bone and new vertical bone can grow at its top. Therefore, it can be used even where mini-implants are no option.

Moreover, this study was designed to verify whether rhBMP-2 and VEGF work synergistically in order to promote vertical bone growth. Former in vitro and in vivo studies suggested a positive effect on bone growth by using the combination of VEGF and rhBMP-2 [44, 48, 51] but it has not been tested for vertical bone augmentation in vivo yet.

## Methods

### Implants and scaffolds

The dental implants used were 11 mm in length and 3.5 mm in diameter (Nobel Replace Straight NP, Nobel Biocare, Goeteborg, Sweden). A disc–shaped scaffold of insoluble collagenous bone matrix (ICBM, 10 mm of diameter in total, inner diameter 3.5 mm, 5 mm of height and with a volume of 345 mm^3^) was designed to exactly fit the coronal part of the implant whilst lying above the local bone. 138 μg rhBMP-2 (provided by Prof. Dr. W. Sebald, Wuerzburg, Germany) and 18.4 μg VEGF (Recombinant Human VEGF165 #293-VE-050, R&D Systems Europe, Ltd., Abington, United Kingdom) were applied onto the ICBM-Carrier.

### Animal study

Twelve mini pigs (average body weight 66 kg of both genders) were treated in this study. They were kept according to official standards. The usage of mini pigs has been approved by the Animal Ethics Committee of the University of Duesseldorf.

For all surgical procedures animals underwent general anaesthesia. Preoperatively the animals were sedated by the use of 10 mg/kgKG Ketamin (Ketavet®, Pfizer, Karlsruhe, Germany) and 5 mg/kgKG Azaperon (Stresnil®, Janssen-Cilag, Neuss, Germany). General anaesthesia was induced by Thiopental (Thiopental inresa®, Inresa Arzneimittel GmbH, Freiburg, Germany), followed by endotracheal intubation. Endotracheal anaesthesia was performed by using Isofluran for induction and maintenance. In addition, for intraoperative pain management 0.5 ml Piritramid (Dipidolor®, Janssen-Cilag, Neuss, Germany) and Articainhydrochloride (Ultracain® DS, 1:200.000, Aventis, Frankfurt, Germany) were used. For dental extraction, the oral cavity was cleaned by antiseptic mouthwash (Hexoral®, Pfizer, Karlsruhe, Germany), gingival margins were cut and relieving incisions were made. The mucoperiosteal flap was raised and the two premolars and the first molar teeth were removed. Bone ridges were flattened, plastic reconstruction of the extraction site and subsequent saliva proof wound closure was performed (Vicryl® 2/0, Ethicon GmbH, Norderstedt, Germany).

After 3 months 108 dental implants were inserted into the mandible (72 implants) and the tibia (36 implants) of the 12 mini pigs. All surgery was performed under sterile conditions in a veterinary operating theatre. The intraorally, gingival margins were cut, relieving incisions were made and a mucoperiosteal flap was elevated. Three implants on each side of the mandible were inserted according to manufacturer’s instructions. They were implanted overlapping the residing bone by 5 mm. Depending on the experimental group the implant was either allowed to heal by itself (group 1), covered with ICBM (group 2), covered with ICBM containing rhBMP-2 (group 3), or covered by ICBM containing rhBMP-2 and VEGF (group 4). The periosteum was incised allowing the mucoperiosteal flap to tensionless cover the implanted area. Saliva-proof wound closure was performed by interrupted sutures using Vicryl 2.0. At the tibia, bone was exposed over a distance of 10 cm by atraumatic preparation towards the bone after incision of the skin and the subcutaneous tissue. The periosteum was incised and implants were inserted following manufacturer’s instructions according to test groups. A multi-layered wound closure was performed by interrupted sutures using Vicryl 2.0.

During surgery and for 3 days postoperatively the mini pigs received oral antibiotic coverage (Amoxicillin 10 mg/kg body weight, Duphamox LA®, Fort Dodge, Wuerselen, Germany) and decongestant medication (Carproven p.o. 4.4 mg/kg body weight, Rimadyl®, Pfizer, Karlsruhe, Germany). 4 of the animals were euthanized by pentobarbital overdosing (Eutha 77® ad us. vet, Essex Pharma, Muenchen, Germany) after 2, 4 and 12 weeks, respectively. Afterwards, tibial and mandibular block specimens were harvested.

### Histological and histomorphometric preparation

Specimens were fixed in 4% formaldehyde, dehydrated, embedded in Technovit® 7200 VLC (Heraeus Kulzer GmbH, Wehrheim, Germany), and polymerized. Utilizing the cutting-grinding technique according to Donath, longitudinal sections were grounded to about 20–40 μm for conventional microscopy (EXACT-Apparatebau, Norderstedt, Germany). Samples were stained according to manufacturer’s protocols with Masson-Trichrom-Goldner and toluidine blue. To examine, evaluate, and photograph the specimens light microscopes (Leica DM 5000B, Leica Microsystems, Wetzlar, Germay, and Olympus BX50, Olympus, Hamburg, Germany) equipped with a microscopic high resolution camera (Leica DFC 40020C, Leica Wetzlar, Germany) were used. With the help of image measuring software (Cell D®, Soft Imaging System, Muenster, Germany and Leica Application-Suite LA5 V3.7 Leica Microsystems Imaging Solutions Ltd., 2003, Wetzlar, Germany) four main measurements were performed: Bone-to implant-contact % (BIC%) was measured by manually marking the areas in which bone was attached to the surface of the implant using 40-fold magnification. The result was then divided by the measured length of the implant including windings and multiplied by 100. Bone-volume-density % (BVD%) was measured marking a distinct 2mm^2^ area, one side of the cube touching the residing bone and one side touching the implant. Within the cube the measurement software (colour coded and manually adjusted beforehand) detected the percentage of newly generated young bone. Furthermore, the length of the defect and the most coronal bone-implant-contact were measured. Using those two parameters, the amount of newly generated periimplant vertical bone (periimplant vertical bone gain = PVBG) could be calculated. Moreover, by dividing the measured most coronal bone formation by the length of the defect, vertical bone gain (VBG) in total was calculated.

### Statistical analysis

Seventy seven implants were used for evaluation (51 Mandible, 26 Tibia). Twenty seven implants were separated for a different study. Four specimens were lost during preparation process. A Kolmogorov-Smirnov-Test was used to detect normal distribution of values. A linear regression analysis was executed to detect dependencies. *P* < 0.05 was set for a significant divergence, whereas *p* > 0,01 was considered to be highly significant. Calculations were performed by the use of SPSS 21 for Windows (SPSS Inc., Chicago, IL, USA) and with the help of Dr. Wolfgang Kaisers (CBiBs, Heinrich-Heine-University Duesseldorf, Deutschland).

## Results

**Fig. 1:**
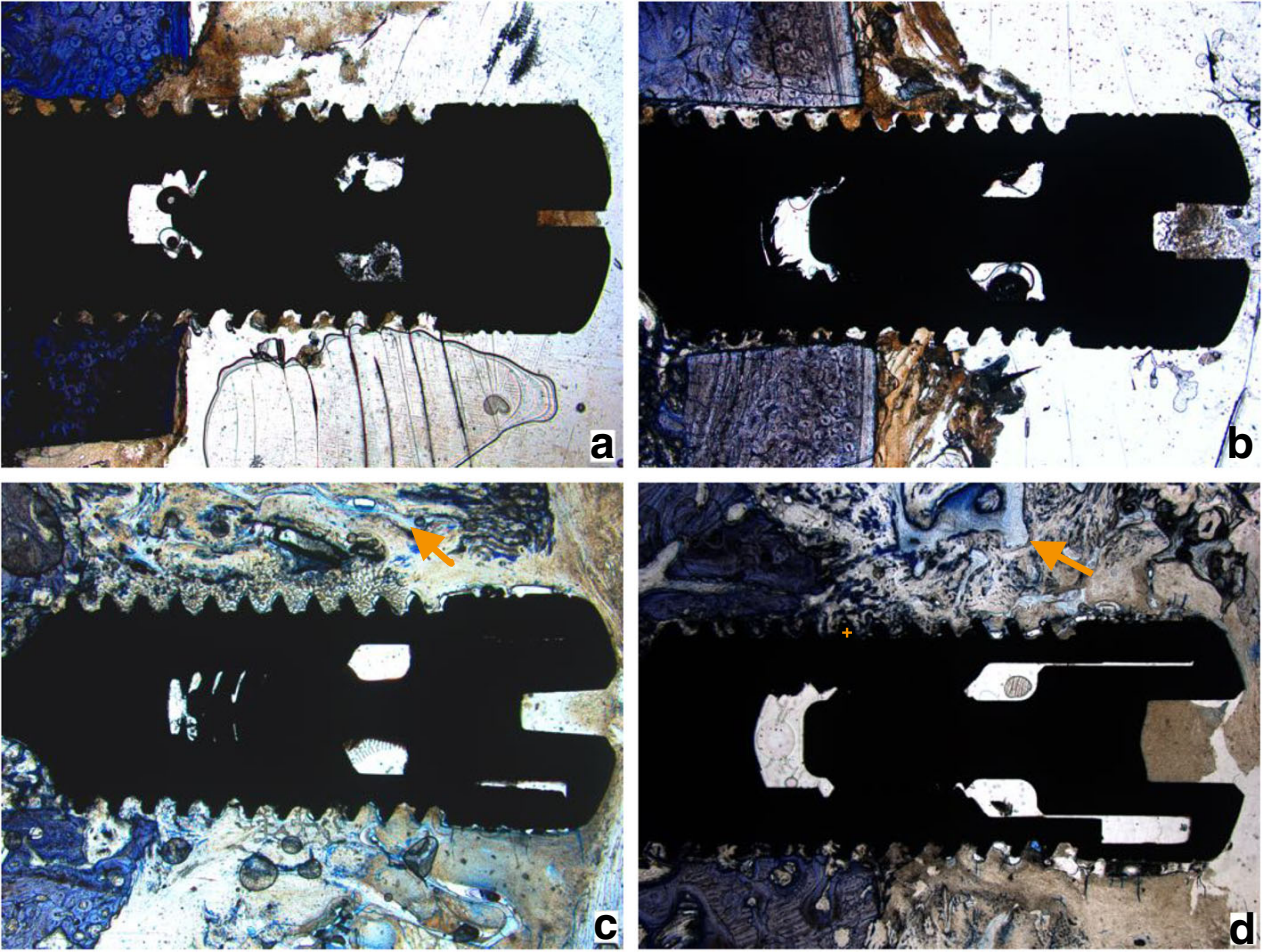
Histological results after 2 weeks: **a** Implant by itself, **b** Implant with ICBM-Carrier, **c** Implant with ICBM-Carrier and rhBMP-2, **d** Implant with ICBM, rhBMP-2 and VEGF, + indicates new bone formation around the windings of the implant, → indicates islands of newly formed osteoid matrix

**Fig. 2:**
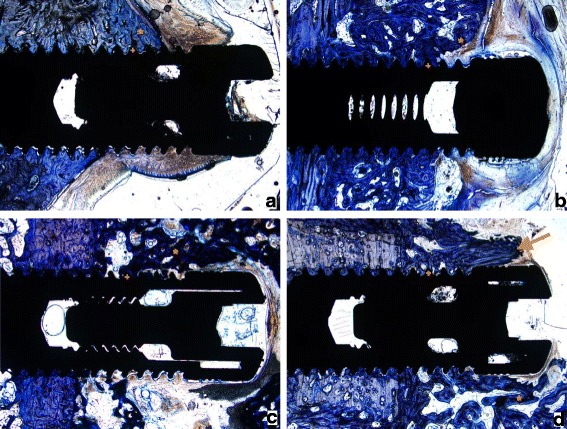
Histological results after 12 weeks: **a** Implant by itself, **b** Implant with ICBM-Carrier, **c** Implant with ICBM-Carrier and rhBMP-2, **d** Implant with ICBM, rhBMP-2 and VEGF, + indicates new bone formation around the winding of the implant, * indicates newly formed trabeculae of woven bone, → indicates lamellar bone

Light microscopic examinations of the control group and the ICBM group showed no or very little new bone formation after 2 weeks (Fig. 1). Around implants covered by ICBM + BMP-2 and ICBM + BMP-2 + VEGF islands of osteoid could be found within the area of the ICBM-scaffold. After 4 weeks in all groups new bone was formed. In the ICBM + BMP-2 + VEGF group new bone grew circumferentially around the implant presenting trabecular bone and primary bone marrow. After 12 weeks of healing all groups presented trabecular bone formation and primary bone marrow. Areas of dense lamellar bone showed around implants with ICBM + BMP-2 + VEGF (Fig. 2).

**Fig. 3:**
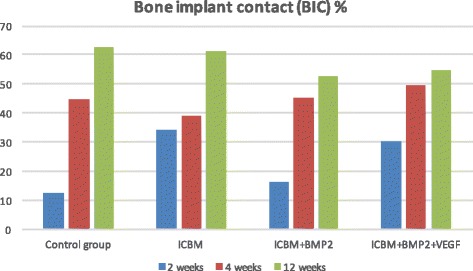
Average BIC% during research period

**Fig. 4:**
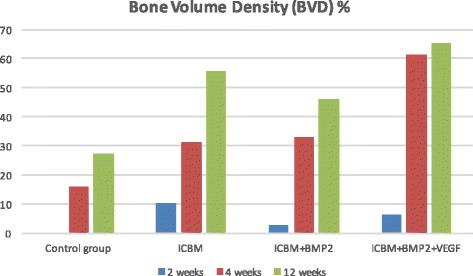
Average BVD% during research period

**Fig. 5:**
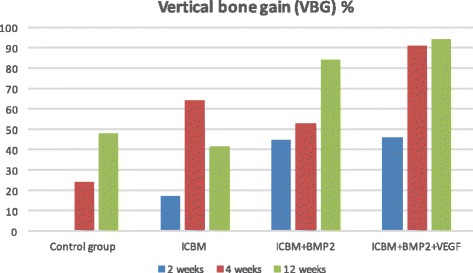
Average vertical bone gain during research period

**Fig. 6:**
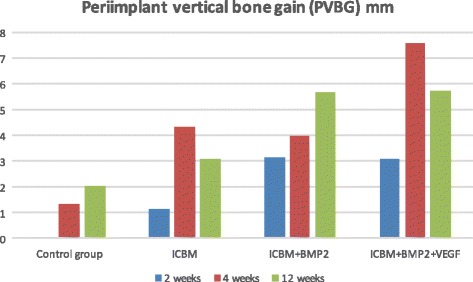
Average periimplant vertical bone gain during research period

Slight bone-to implant-contact (Fig. 3) was noticeable in all groups after 2 weeks (Table 1). ICBM and ICBM + BMP-2 + VEGF covered implants showed the highest mean values (ICBM 34.4% (±8.9%), ICBM + BMP-2 + VEGF 30.2% (±11.7%)). The other two groups showed less bone-to implant-contact. In terms of bone-volume density the ICBM group started off with the highest value of 10.3% (±7.9%) whereas there was no BVD measurable in the control group. Vertical bone gain (VBG) showed to be almost the same in ICBM + BMP-2 (45% (±40%)) and ICBM + BMP-2+ VEGF (46% (±54%)). In the ICBM group an average of (17% (±8%)) was calculated. There was no VBG measurable in specimens containing only the implant. In terms of periimplant vertical bone ICBM + BMP-2 reached 3.1 mm (±3.1 mm) and ICBM + BMP-2 + VEGF came to 3.1 mm (±3 mm) after 2 weeks. Upon 4 weeks of the healing mean BIC percentages varied from 38.9% (±10.37%) in the ICBM group to 49.3% (±19.7%) in the ICBM + BMP-2 + VEGF group. BVD percentages varied even more. The average BVD (Fig. 4) regarding the implant on its own was 15.8% (±22.57%). A measured negative value (below zero) equalled bone resorption. ICBM + BMP-2 + VEGF came to 61.3% (±14.5). Vertical bone gain (Fig. 5) shows a similar distribution. Mean VBG in the control group was measured 24% (±29%), whereas ICBM + BMP-2 + VEGF group averages measured ICBM + BMP-2 + VEGF 91% (±40%). Periimplant vertical bone gain (Fig. 6) sums up to 1.34 mm (±2.3 mm) in the control group, to 4.34 mm (±4.5 mm) in the ICBM-group, 4 mm (±3.1 mm) in the ICBM + BMP-2 group and 7.6 mm (±3.01 mm) in the ICBM + BMP-2 + VEGF group. After 12 weeks mean BIC percentages for control and ICBM groups were almost equal accounting for 62.7%, (±15.1%) for the implant alone and 61.3% (±16.2%) for ICBM. ICBM + BMP-2 and ICBM + BMP-2 + VEGF stayed behind. Implants covered by ICBM + BMP-2 + VEGF showed the highest percentages (65% (±20.5%)).

**Table 1:** Summary of measured mean values for Bone-implant-contact, Bone-volume-density, Vertical-bone-gain and periimplant-vertical-bone gain

	Control group	ICBM	ICBM + BMP-2	ICBM + BMP-2 + VEGF
BIC%				
2 weeks	12.5	34.4	16.3	30.2
4 weeks	44.6	38.9	45	49.3
12 weeks	62.7	61.3	52.7	54.7
BVD%				
2 weeks	0	10.3	3	6.1
4 weeks	15.8	31.4	33.1	61.3
12 weeks	27.5	55.8	46.1	65
VBG%				
2 weeks	0	17	45	48
4 weeks	24	64	53	91
12 weeks	48	42	84	94
PVBG (mm)				
2 weeks	0	1.1	3.1	3.1
4 weeks	1.3	4.3	4	7.6
12 weeks	2.1	3.1	5.7	5.8

The lowest values were found in the control group (27.5% (±47.6%)). Vertical bone gain (VBG) in specimens containing only the implant added up to 48% (±83%). Those implants being covered by ICBM + BMP-2 + VEGF added up to 94% (±54%). In terms of vertical bone gain around the implant (PVBG) ICBM + BMP-2 (5.8 mm (±3.2 mm)) and ICBM + BMP-2 + VEGF (5.6 mm (±3.8 mm)) groups showed similar values after 12 weeks and the control group displayed (2.1 mm (±3.6 mm)) of gained bone height (Table 1). Statistically significant differences could be seen in vertical bone gain between control and ICBM + BMP-2 + VEGF groups (*p* = 0.0158). Highly significant differences were detected in bone-volume density (*p* = 0.0011) and in periimplant vertical bone gain (*p* = 0.0018) between the control and the ICBM + BMP-2 + VEGF group.

With the intention of using as few animals as possible, implants were inserted into the tibia and into the lower jaw. Although bone recovery differs and the exposure to bacteria in the oral cavity is missing, the tibia equals the lower jaw best in terms of bone size and volume. Furthermore, oral hygiene cannot be managed in mini-pigs, resulting in an increased loss of implants. Overall, implants inserted into the tibia showed higher values than implants inserted into the lower jaw but in order to get a reliable statistical outcome results could not be analysed separately (Table 2).

**Table 2:** Summary of mean values for Bone-implant-contact, Bone-volume-density, Vertical-bone-gain and periimplant-vertical-bone gain divided into mandible and tibia

	Control Group		ICBM-Carrier		ICBM + BMP-2		ICBM + BMP-2 + VEGF	
	Mandible	Tibia	Mandible	Tibia	Mandible	Tibia	Mandible	Tibia
BIC%								
2 weeks	no data	12.5	no data	34.4	18.4	14.1	42	10.7
4 weeks	44.6	no data	39.6	37.4	40.3	73.3	43.8	60.4
12 weeks	68.1	51.8	43.3	70.3	49.1	57.2	49.4	65.2
BVD%								
2 weeks	no data	0	no data	10.3	5.9	0	9.7	0
4 weeks	15.8	no data	13.5	67.1	1.1	68.6	58.5	66.9
12 weeks	0	82.4	2.1	82.7	42.9	50	59.7	75.6
VBG%								
2 weeks	no data	0	no data	17.3	40	35.1	46.1	44.9
4 weeks	24	no data	30.1	137.2	41.8	117.5	66.1	139.8
12 weeks	0	143.4	0.6	62.6	38.4	141	66.8	148.8
PVBG (mm)								
2 weeks	no data	0	no data	1.1	4.4	1.9	3.2	3.1
4 weeks	1.3	no data	2.5	8	3.5	6.7	5.9	10.9
12 weeks	0	6.2	0.1	4.9	3.1	9	5	7.2

## Discussion

All individual parts of this study were subject of former research studies. RhBMP-2 has proven to enhance bone regeneration adjacent to dental implants [52]. VEGF is known to induce angiogenesis [45, 46, 53]. Bai et al. [54] and Lin et al. [55] tested the combination of rhBMP-2 and VEGF, which seems to accelerate bone healing. Different carrier materials [2, 5, 34, 56, 57] have been examined but the ultimate carrier is yet to be found. ICBM itself has shown bone regenerative capacity in combination with embryonal stem cells [58, 59]. A combination of all of those materials in vivo, however, has never been described before.

The surface of dental implants is decisive for chemotaxis and cell activation [60]. Sand-blasted and acid etched surfaces are particularly useful for reliable osseointegration of dental implants [6, 61, 62]. Titan implants with sand-blasted and acid etched surfaces were used as control group in this study. During the 12 week-period of this study linear bone growth could be detected. To release cytokines at implantation site, a carrier system is needed [63]. Ideally, the carrier emits cytokines continuously over a longer period and simultaneously serves as a stable scaffold for immigrating cells. Giesenhagen harvested disc-shaped autogenous bone blocks of the chin and placed them around the coronal part of the implant. He found them to be particularly helpful in regeneration of three-dimensional vertical bone defects [64]. Carrel et al. 3D printed a tricalciumphosphate and hydroxyapatite scaffold to enhance three - dimensional bone augmentation [57]. The meta-analysis by Troeltzsch et al. indicated that bone blocks significantly enhanced horizontal augmentation in comparison to particulate materials. In terms of vertical augmentation beneficial results depended on the origin of the bone block material [17]. In this study disc-shaped collagenous scaffolds (ICBM carrier) were used fitting precisely around the coronal part of the implants. Results showed that the ICBM alone seems to accelerate periimplant bone regeneration. After 2 weeks, implants covered with ICBM reached higher values in every measurement in comparison to the control group. After 12 weeks, apart from BVD which was higher than in the control group (BVD Control group after 2 weeks 27.5% and BVD ICBM-after 2 weeks 55.8%), no or little difference could be seen. This might be due to collagenous structures of the ICBM acting as a scaffold for osteoblasts to migrate and generate new bone. Over time reabsorbing processes around the implant take place. Therefore, ICBM alone only seems to provide an advantage in vertical bone growth during the first 4 weeks of implantation.

Recombinant rhBMP-2 enhances bone regeneration [2, 65, 66]. RhBMP-2 initiates differentiation of embryonal stem cells into chondroblasts, osteoblasts or adipocytes. While lower concentrations of rhBMP-2 are assumed to boost initial chondrification higher concentrations are supposed to enhance osteogenesis [67]. Over the entire examination period ICBM + BMP-2 showed lower BIC% values compared to the implant alone. However, measured values of VBG, PVBG and BVD were higher compared to the control group. A study of Jones et al. showed a similar outcome. RhBMP-2 induced periimplant bone regeneration showed a higher BIC%, more new bone formation and a better filling of the defect after 4 weeks but after 12 weeks no difference was detectable anymore [2]. This effect was thought to be due to negative feedback mechanisms of the rhBMP-2 signalling cascade putting a break on new bone formation. Nevertheless, given that case, VBG, PVBG and BVD would decrease as well. A different explanation could be a lack of angiogenesis to support rhBMP-2-accelerated bone growth. Matsubara et al. stated that bone formation can be delayed in order to ensure sufficient vascularization [68]. This could explain the reduced growth rate after 4 weeks. The carrier-complex itself might also be the reason for a reduced BIC in comparison to the control group. Directly around the implant concentrations of rhBMP-2 might have been lower than at the centre of the ring (approximately 2 mm away from implant surface). Three possible explanations might be: 1. more rhBMP-2 was available in these areas, 2. less rhBMP-2 was necessary for sufficient bone growth or 3. rhBMP-2 could be used more effectively. In a similar study Refaat et al. used ICBM-Carriers and rhBMP-2 for spinal fusion. They used much lower doses of rhBMP-2 (2 μg and 10 μg). RhBMP-2 was released by 100% within 20 days, irrespective of the dose provided [69]. If these results were to be transferred to this study, a positive effect of BMP2 would only be possible in the first 4 weeks after implantation.

Angiogenesis is crucial for bone formation. Local application of VEGF prolongs angiogenesis at defect sites and therefore reinforces osteogenesis [46, 47]. For craniofacial and mandibular bone in particular, sufficient amounts of VEGF are necessary for ossification [48]. Peng et al. examined the synergistic potential of rhBMP-2 and VEGF. Outcomes described a dependant relationship between these two [70]. Jiang et al. found increased bone generation combining VEGF and rhBMP-2 compared to only using one of them. Furthermore, they stated VEGF to be the determining factor of enhancement in bone formation in vitro [44]. The present in vivo study supports those findings. Measured results of ICBM + BMP-2 + VEGF were similar or higher compared to ICBM + BMP-2. Especially bone volume density and vertical bone gain were substantially higher after 4 and 12 weeks of the healing process compared to ICBM + BMP-2. There might even be a higher dependency of rhBMP-2 and VEGF than formerly thought. According to Matsubara et al. vascular endothelial cells and muscle cells are supposed to be the primary source of rhBMP-2 expression during osteogenesis. Results of their studies showed that mainly blood vessels release rhBMP-2 and mesenchymal stem cells synthesize VEGF [68]. In this study ICBM + BMP-2 + VEGF showed a rapid bone growth which wears off after 4 weeks in comparison to implants alone. These results indicate along with other studies [55] that prolonged artificial high levels of VEGF over the physiological period of 31 days do not improve bone healing. This might be due to decreasing VEGF and rhBMP-2 levels according to consumption over time. Kumar et al. solved this problem by using stem cells producing VEGF and rhBMP-2 [47]. To sum up, the combination of ICBM + BMP-2 + VEGF significantly outranks implants alone in terms of bone volume density, vertical bone gain and periimplant vertical bone gain. Only short term improvement of bone to implant contact could be seen.

This study being an animal study, the number of samples had to be held small. Therefore, values varied significantly. In order to get more reliable results further research has yet to follow.

## Conclusion

Results of this study suggest that the combination of rhBMP-2 and VEGF applied locally by using a collagenous carrier enhances vertical bone generation around the implant in vivo. In defect areas high quality and quantity of bone could be detected in comparison to implants alone, implants covered with ICBM and implants covered with ICBM+ rhBMP-2. A one stage procedure and the use of a collagenous disc-shaped scaffold reduced the number of surgery to a minimum, eliminated donor site morbidity and maintained satisfactory volume stability as there was only little resorption over time.

Further investigations have yet to show how rhBMP-2 and VEGF can be used in a safe and predictable way in order to use them for bone regeneration in clinical routines.

## Abbreviations

GBR: Guided bone regeneration
ICBM: Insoluble collagenous bone matrix
rhBMP-2: Recombinant human bone morphogenetic protein-2
VEGF: Vascular endothelial growth factor
